# Zebrafish as a Model for Germ Cell Regeneration

**DOI:** 10.3389/fcell.2021.685001

**Published:** 2021-07-22

**Authors:** Zigang Cao, Qifen Yang, Lingfei Luo

**Affiliations:** ^1^Jiangxi Key Laboratory of Organ Developmental Biology, College of Life Sciences, Jinggangshan University, Ji’an, China; ^2^Institute of Developmental Biology and Regenerative Medicine, Southwest University, Chongqing, China

**Keywords:** zebrafish, germ cell, regeneration, germline stem cell, *nanos2*

## Abstract

Germ cell acts as a link between transfer of genetic information and process of species evolution. Defects or malformations of germ cells can lead to infertility or tumors. Germ cell regeneration is one of the effective ways to treat the infertility. Therefore, it is of great scientific and clinical interests to dissect the cellular and molecular mechanisms underlying germ cell regeneration. Progress have already been achieved in germ cell regeneration using model organisms for decades. However, key open issues regarding the underpinning mechanisms still remain poorly understood. Zebrafish is well known for its powerful regenerative capacity to regenerate various tissues and organs. Recently, advances in genomics, genetics, microscopy, and single cell technologies have made zebrafish an attractive model to study germ cell development and regeneration. Here we review recent technologies for the study of germ cell regeneration in zebrafish, highlight the potential of germline stem cells (GSCs) in the contribution to reproductive system regeneration, and discuss the *nanos*. Wnt signaling and germ cell-specific factors involved in the regulation of germ cell regeneration.

## Introduction

Infertility is estimated to affect more than 186 million people in the world (Inhorn and Patrizio, 2015). In the next decade, infertility is assessed to remain a highly common global disease. The overall prevalence of infertility was estimated to be 8%–12% (Ombelet et al.,2008a,b; Inhorn and Patrizio, 2015). However, the infertility rate is much higher in some regions of the world, reaching about 30% (Nachtigall, 2006; Inhorn and Patrizio, 2015). Defects or malformations of germ cells can lead to infertility or tumors. Germ cell regeneration is one of the effective ways to treat the infertility. Therefore, using model organisms to dissect the mechanisms underlying germ cell regeneration has great scientific and clinical significance.

Animals such as teleost fish and amphibians possess the abilities to regenerate various tissues and organs, while other animals including mammals are limited in regeneration. Zebrafish (*Danio rerio*) has remarkable regenerative capabilities and becomes one of the most widely used vertebrate models for regenerative studies. Zebrafish is capable of regenerating a range of tissues including fin (Shibata et al., 2016; Cao et al., 2021), heart (Poss et al., 2002), spinal cord (Mokalled et al., 2016), brain (Lucini et al., 2018), liver (He et al., 2014), hair cells (Thomas and Raible, 2019), kidney (Reimschuessel, 2001), and retina (Vihtelic and Hyde, 2000). Although regeneration studies mainly focus on these somatic tissues, recent studies about reproductive system regeneration attract much interest. Progress have already been achieved in reproductive regeneration using model organisms for decades. However, key open issues regarding the underpinning mechanisms still remain poorly understood.

Zebrafish germ cell development is conserved among animals. Various factors and pathways regulating germ cell development in mammals were found and have similar functions in zebrafish. However, in comparison with mammals, zebrafish maintain high fertility throughout their lives due to ovarian GSCs driving continuous production of eggs. Recently, evidences show that zebrafish reproductive systems have regenerative capabilities and is capable of producing new germ cells after injuries (White et al., 2011; Cao et al., 2019). Moreover, with the continuous advancement of genomics, genetics, microscopy, and single cell technologies, zebrafish becomes an attractive model to study germ cell development and regeneration. Although it is commonly believed that human (or mice) lacks GSCs, the regulatory mechanisms underlying germ cell development and regeneration are conserved in vertebrates. Thus, using zebrafish to dissect the mechanisms underlying germ cell regeneration will be helpful for understanding the mechanism of infertility in human and providing inspiration in mammalians reproductive disease. In this review, we summarize recent technologies and tools for the study of germ cell regeneration and focus on the cellular and molecular mechanisms underlying germ cell regeneration in zebrafish.

## Tools for Studying Germ Cell Regeneration

Several available tools have been applied in zebrafish to ablate germ cells or damage gonad for regeneration studies. These applications range from killing most of the targeted cell types to surgical removal of partial organs, as illustrated in Table 1, and are mainly divided into three types. The extent of germ cell elimination leads to difference regenerative results.

**TABLE 1:** Tools for germ cell elimination in teleost fish.

Type of germ cell elimination	Species/Sexuality	Treatment or genetic manipulation	Time period of treatment	Extent of ablation	Characteristics/Conclusion	References
Genetic ablation	Zebrafish/female	*Tg(zp:egfp-NTR)*/5 mM MTZ for 14 d	28 dpf	All of germ cell elimination	Oocyte apoptosis/no regeneration	Hu et al. (2010)
	Zebrafish/female	*Tg*(*zpc:g4vp16/uas:nfsb-mcherry*)/5 mM MTZ for 14 d	8–12 mpf	Most of oocytes elimination	Regenerated by germ cell proliferation	White et al. (2011)
	Zebrafish/female	*Tg(ziwi:CFP-NTR*)/3 times×16 h in 10	5 mpf	Most of germ cell elimination	Sex-reversal/fertile male	Dranow et al. (2013)
	Zebrafish/female	*Tg*(*vasa:dendra2-NTR*)/8 mM MTZ for 7 d	3 mpf	All of GSCs elimination	Sex-reversal/infertile male	Cao et al. (2019)
	Zebrafish/female	*Tg*(*aos/asp/odf/sam: egfp-ntr*)/5 mM MTZ for 14 d	28 dpf	All of germ cell elimination	Infertile/no regeneration	Hsu et al. (2010)
Chemically inducing germ cell damage	Zebrafish/male	busulfan treatment for 12 d	Adult	Mitotic germ cell elimination	Regenerated by GSCs proliferation	Nóbrega et al. (2010)
	Medaka/female	busulfan treatment for 1 week	3–4 mpf	Mitotic germ cell elimination	Regenerated by GSCs proliferation	Nakamura et al. (2010)
Surgical injuries	Zebrafish/female	ovariectomy	50–90 dpf	Most of one side ovary removal	Regenerated by GSCs proliferation	Cao et al. (2019)
	Chinese grass carp/female	ovariectomy	Adult	No description	Regenerated by remaining ovarian tissue	Underwood et al. (1986)
	Rainbow trout/female	ovariectomy	Adult	No description	Regenerated by remaining ovarian tissue	Kersten et al. (2001)
	Betta Splendens/female	ovariectomy	Adult	All of ovarian tissues removal	Sex-reversal	Lowe et al. (1975)

### Genetic Ablation

In the past two decades, genetic ablation become widely used in organ regeneration for ablation of the targeted cell types. The basic principle of this approach is the generation of transgenic line driving tissue- or cell type-specific protein expression, which induces the targeted tissues or cell death. Several types of these proteins have been developed, including KillerRed, bacterial toxin Kid and diphtheria toxin A chain (Kurita et al., 2003; Bulina et al., 2006; Wang et al., 2011). However, the most widely used approach is the transgenic lines combining with chemicals, which can conditionally induce the toxic factors. This approach utilizes nitroreductase (NTR), a bacterial enzyme that catalyzes its substrate metronidazole (MTZ), a non-toxic prodrug, into cytotoxic metabolite that causes DNA crosslink, finally inducing cell death. This technique has been used for targeted cell ablation in cancer therapy (Connors, 1995; Searle et al., 2004). By the transgenic approaches, a cell or tissue-specific promoter driving NTR is expressed in certain cells and tissues and then converts the MTZ into the cytotoxic metabolite only in NTR-expressing cells, finally leading to ablation of targeted cells or tissues (Curado et al., 2008). With fluorescence microscopy and fusion protein engineering, the NTR-fluorescence fusion protein is able to monitor and visualize the progress of cell ablation in a real-time manner, making it easier to optimize the conditions of MTZ treatment (Chen et al., 2019; He et al., 2019).

**FIGURE 1:**
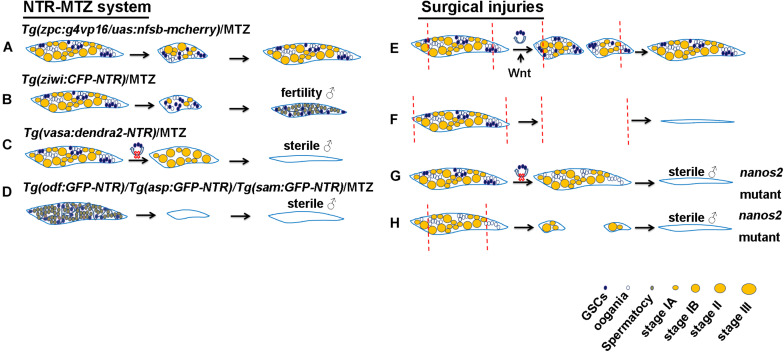
Schematic of genetic ablation by NTR-MTZ system and ovarian surgical injuries in zebrafish. **(A)** All of GSCs in the *Tg(vasa:dendra2-NTR)* transgenic background females were ablated by MTZ and they finally reverted into the sterile males. **(B)** A large number of oocytes in the *Tg(zpc:g4vp16/uas:nfsb-mcherry)* transgenic background females were ablated by MTZ and were completely regenerated. **(C)** Most of germ cells in the *Tg(ziwi:CFP-NTR)* transgenic background females were ablated by MTZ and they finally reverted into the fertile males. **(D)** Germ cells in the *Tg(asp:GPF-NTR)*, *Tg(sam:GPF-NTR)* and *Tg(odf:GPF-NTR)* transgenic background males were ablated by MTZ and they finally develop into the sterile males. **(E)** Most of the removed ovarian tissues were regenerated due to the proliferation and differentiation of GSCs and Wnt signaling regulated this process. **(F)** Regeneration failed to occur after all of ovarian tissues amputation. **(G)** All of GSCs were lost in *nanos2* mutant ovaries and caused sex-reversal. **(H)** Ovaries in *nanos2* mutants failed to regenerate due to loss of GSCs. Red dotted lines represent the distance of ovarian resection.

To construct transgenic fish that have specific expression of NTR in germ cells, promoters of the germ cell-specific genes, *vasa* and *ziwi*, the oocyte-specific gene, *zpc*, as well as the testis-specific genes, *asp*, *sam*, and *odf*, were employed to drive NTR-fluorescence fusion protein expression (Hu et al., 2010; Hsu et al., 2010; Dranow et al., 2013; Cao et al., 2019). These studies reported that using 5 mM MTZ to treat the *Tg(zp:GFP-NTR)* transgenic line zebrafish females at 28 day post-fertilization (dpf) for 2 weeks caused infertility due to complete apoptosis of their germ cells (Hu et al., 2010), whereas other study used the same method to treat the adult *Tg(zpc:g4vp16/uas:nfsb-mcherry)* transgenic background females and found that a large number of oocytes were killed, but 1 month later the ovaries could recover completely to regain their reproductive functions (Figure 1A) (White et al., 2011). Furthermore, the *Tg(ziwi:CFP-NTR)* transgenic background females reverted into the fertile males after most of ovarian gem cells were ablated by MTZ treatment (Figure 1B) (Dranow et al., 2013, 2016), while the *Tg(vasa:dendra2-NTR)* transgenic line females failed to regenerate and finally reverted into the infertile males when the early stage germ cells were ablated by MTZ (Figure 1C) (Cao et al., 2019). Additionally, MTZ was able to induce male infertility by targeted germ cells ablation in the testes of *Tg(asp:GPF-NTR)*, *Tg(sam:GPF-NTR)* and *Tg(odf:GPF-NTR)* transgenic background zebrafish (Figure 1D) (Hsu et al., 2010).

### Chemically Induced Germ Cell Damage

Besides genetic ablation, gonadal injuries or germ cell elimination can be induced by chemical compounds. Busulfan, an anticancer drug, can be used to kill proliferating germ cells. Adult male zebrafish treated with busulfan by intraperitoneal injection had a significant reduction in the number of germ cells, but the germ cells were subsequently regenerated as a result of GSC proliferation (Nóbrega et al., 2010). Similarly, adult medaka females treated with busulfan by intraperitoneal injection for 1 week had only a handful of GSCs in their ovaries, but after 3 months, the number of ovarian germ cells recovered to normal levels, indicating that proliferating ovarian GSCs replenishes the ablated germ cells (Nakamura et al., 2010). However, this method has some disadvantages, such as a less uniform damage and off-target effects (Strahle and Grabher, 2010).

### Surgical Injuries

The classical method for organ regeneration is surgical injury and tissue resection or amputation is one of the most common surgical methods. This technique is widely used for fin, heart and liver (White et al., 1994; Burkhardt-Holm et al., 1999; Poss et al., 2002). Reproductive injuries of fish were also reported. For example, attempts to make Chinese grass carp infertile through ovarian surgical resection have not been successful due to ovarian regeneration (Underwood et al., 1986). Similar experiments in rainbow trout also showed ovarian regeneration following ovarian resection due to proliferation of the remaining gonadal tissues, but complete ovarian resection leads to infertility (Kersten et al., 2001). In zebrafish, nearly 95% of ovarian tissues on one side were resected and completely regenerated in one month and reproductive abilities were also resumed (Figure 1E), suggesting the powerful regenerative abilities of zebrafish ovaries, but regeneration failed to occur when all of left ovarian tissues were amputated (Figure 1F) (Cao et al., 2019). Similarly, complete removal of the ovaries can cause a lack of gonadal regeneration in Betta splendens and subsequent female-to-male reversal (Lowe et al., 1975). In addition to teleost fish, gonads of some higher vertebrates were reported to be capable of regeneration. For example, Adult axolotl salamanders were also able to regenerate the amputated tissues after hemi-ovariectomy, and resumed reproductive ability after a healing period of 5 months. Oogonial stem cells were activated and contributed to regeneration (Erler et al., 2017). Furthermore, some lower invertebrates such as planarian can completely regenerate its reproductive system with a small piece of tissues losing gonad, and the conserved gene *nanos* plays important roles in the process of regeneration (Wang et al., 2007).

## Cellular and Molecular Mechanisms Underlying Germ Cell Regeneration

Building a model of injury and regeneration is the first step to study regenerative biology or medicine using all these tools. The most important content of regenerative research is to reveal the cellular and molecular mechanisms underlying organ regeneration. The following section aims to provide an overview of cellular sources that contribute to germ cell and gonadal regeneration, and discuss the key factors and signaling pathways involved in the regulation of germ cell regeneration.

### Cellular Sources

Depending on the nature or degree of injuries involved, a number of potential sources contribute to newly generated cells. For example, when liver damage is not too severe, proliferation of remaining hepatocytes contributes to regeneration (Michalopoulos, 2007). However, regeneration occurs after severe hepatocyte loss via the transdifferentiation of biliary epithelial cells into hepatocytes in the liver (He et al., 2014). Cellular origin underlying organ or tissues regeneration mainly include proliferation of existing cell types (Poss et al., 2002; Sadler et al., 2007), cellular dedifferentiation (Stewart and Stankunas, 2012), cellular transdifferentiation (He et al., 2014, 2019), and differentiation of stem cells or progenitors (Diep et al., 2011). Many reports suggest that injuries to germ cell or gonadal tissues mainly resolve by the proliferation and differentiation of resident stem cells or progenitors (Nakamura et al., 2010; Erler et al., 2017; Cao et al., 2019).

#### Germline Stem Cells

It is widely accepted that spermatogonial stem cells (SSCs) are consistently present in the animal testes and support spermatogenesis. However, whether the mature ovaries of all animals have GSCs or oogonial stem cells (OSCs) remains to be determined. In invertebrate ovaries, the existence of GSCs has been reported by many articles, and *Drosophila* ovarian GSCs have been a hot topic in scientific research (Cuevas and Spradling, 1998). In vertebrates, GSCs have been identified and labeled by *nanos2* in the mature ovaries of medakas by lineage-tracing experiments (Nakamura et al., 2010). Recent studies provide evidence of GSCs or OSCs in zebrafish mature ovaries (Draper et al., 2007; Wong et al., 2011; Beer and Draper, 2013; Cao et al., 2019). For example, *vasa*-labeled germ cells isolated from adult ovaries and transplanted into sterile host larvae led to the production of zebrafish germline chimeras and restoration of fertility, indicating the presence of mitotically active adult OSCs in zebrafish mature ovaries (Wong et al., 2011). Zebrafish *nanos3* can maintain GSCs and expression of the RNA binding gene *nanos2*, and thus, the female *nanos3* homozygous mutants develop into sterile male fish due to loss of GSCs (Draper et al., 2007; Beer and Draper, 2013). Moreover, we knocked out zebrafish *nanos2*, a marker of GSCs, and found that all of GSCs were absent at 32 dpf, and the female *nanos2* mutants developed into sterile male fish at 75 dpf (Figure 1G) (Cao et al., 2019). These results suggest that similar to medaka, GSCs is specifically labeled and regulated by *nanos2* in zebrafish.

#### Oogenesis in Zebrafish

GSCs stem from primordial germ cells (PGC) and differentiate into oocytes through mitosis and meiosis, which is known as oogenesis. Many genes were reported to be involved in the regulation of oogenesis in zebrafish. *vasa* express specially in germ cells and is required for germ cell differentiation and GSC maintenance in zebrafish (Hartung et al., 2014). *ziwi* and *zili* are also expressed in germ cells and their mutations lead to failure of germ cell maintenance and defect of mature oocytes production in zebrafish (Houwing et al., 2007, 2008). Meiosis defects and loss of germ cells were observed and *tdrd12* deficient fish (Dai et al., 2017). *ca15b* is expressed in PGC and oocytes, and plays an important role in PGC development and oogenesis (Wang et al., 2013). Furthermore, *nanos2* and *nanos3* are required for maintaining oocytes production and GSCs (Draper et al., 2007; Beer and Draper, 2013; Cao et al., 2019).

#### GSCs Contribute to Germ Cell Regeneration

Many evidences suggest that the cellular sources underlying germ cell regeneration is the proliferation and differentiation of GSCs, as illustrated in Table 1. In the NTR-MTZ system for germ cell ablation, since *zpc* only labeled the oocytes, GSCs in the adult *Tg(zpc:g4vp16/uas:nfsb-mcherry)* transgenic background females were not ablated by MTZ. Therefore, their ovaries were able to recovered completely to regain their reproductive function due to the proliferation of the resident GSCs (Figure 1A) (White et al., 2011). *ziwi* can labeled all of germ cells but GSCs in the *Tg(ziwi:CFP-NTR)* transgenic females were not completely ablated by MTZ. Thus, these females reverted into the fertile males (Figure 1B), suggesting that zebrafish ovarian GSCs are bipotential (Dranow et al., 2013, 2016). However, although *vasa* like *ziwi* can label all of germ cells, we ablated all of GSCs in the *Tg(vasa:dendra2-NTR)* by MTZ treatment. These females failed to regain their reproductive functions, indicating that zebrafish ovaries have no regeneration without GSCs (Figure 1C) (Cao et al., 2019). Moreover, our data suggest that GSCs can regenerate the most of removed ovarian tissues in zebrafish after ovariectomy as a result of remaining GSCs proliferation (Figure 1E) (Cao et al., 2019). Other fish ovaries such as Chinese grass carp and Rainbow trout can also regenerate after ovariectomy mainly due to the proliferation of remaining ovarian tissue (Underwood et al., 1986; Kersten et al., 2001). Similarly, newly regenerated germ cells in zebrafish and medaka treated by busulfan originate from the proliferation and differentiation of GSCs (Nakamura et al., 2010; Nóbrega et al., 2010). Furthermore, some reports show that all embryos injected with dead-end MO lose primordial germ cells (PGCs) and develop into sterile males, suggesting that PGCs is essential for fertility in zebrafish (Tzung et al., 2015). Other arguments about the sources of germ cells such as a study reported that regeneration of male germline stem cells occurs in the temperature-sensitive gene stat92E mutants of *Drosophila* due to dedifferentiation of spermatogonial cells. This indicates that germ cells have dedifferentiation phenomena under some certain microenvironment (Brawley and Matunis, 2004). Moreover, Transplantation of testicular or ovarian germ cell aggregates leads to regeneration of spermatogenesis or oogenesis and production of functional sperm or egg in zebrafish (Kawasaki et al., 2010). Thus, germ cell regeneration *in vivo* mainly depends on the proliferation and differentiation of GSCs.

### Factors and Signaling Pathways Regulating Regeneration

In general, some key factors and signaling pathways involved in organ development also participate in the regulation of regeneration. *nanos* family genes and Wnt signaling were involved in germ cell and gonadal development, and were also identified to regulate germ cell and gonadal regeneration. Some germ cell-specific genes such as *dnd*, *piwi1/2*, and *dazl* which are involved in germ cell specification and maintainace may regulate regeneration, as illustrated in Table 2.

**TABLE 2:** Summary of effects of factors contributing to germ cell regeneration.

Factors	Species	Treatment or genetic	Characteristics/Fuctions	References
GSC	Zebrafish	ovariectomy	GSC proliferation in ovary regenration and ablation or defect of GSC lead to failure of regeration	Cao et al. (2019)
*nanos3*	Zebrafish		*nanos3* is essential for PGCs survival, and GSC maintainace. *nanos3* mutations may lead to failure of regeration	Beer and Draper (2013)
*nanos2*	Zebrafish	ovariectomy	Upregulation of *nanos2* expression in regenration and *nanos2* mutations lead to failure of GSC maintainace and regeneration.	Cao et al. (2019)
*dnd*	Zebrafish		Essential for PGCs migration and survival.	Slanchev et al. (2005)
*vasa*	Zebrafish		Required for germ cell differentiation and GSC maintenance. Loss of Vasa may lead to failure of germ cell regeneration.	Hartung et al. (2014)
*ziwi*	Zebrafish		Essential for germ cell maintenance and loss of Ziwi may lead to failure of germ cell regeneration.	Houwing et al. (2008)
*zili*	Zebrafish		Required for germ cell differentiation and meiosis. Loss of Zili may lead to failure of germ cell regeneration.	Houwing et al. (2008)
*dazl*	Zebrafish		Required for GSCs establishment and specification. Loss of Dazl may lead to failure of germ cell regeneration.	Bertho et al. (2021)

#### nanos

Many germ cell-intrinsic factors that regulate the development of GSCs are evolutionarily conserved, especially the functionally conserved *nanos* family genes which play an important role in maintaining GSCs (Forbes and Lehmann, 1998; Sada et al., 2009). The *nanos* homologous gene is a RNA-binding protein that can control translation (Kadyrova et al., 2007). In planarians, *nanos* is expressed specifically in germ cells and essential for germ cell specification and regeneration (Newmark et al., 2008). In *Drosophila*, *nanos* is essential for maintaining ovarian GSCs and its fertility (Forbes and Lehmann, 1998). The *nanos* family genes in vertebrates include three homologous genes, namely, *nanos1*, *nanos2*, and *nanos3*. *nanos1* is mainly expressed in the nervous system, while the other two genes *nanos2* and *nanos3* are both expressed in germ cells (Tsuda et al., 2003; Beer and Draper, 2013). *nanos3* is expressed in migrating PGCs and after birth, is found only in the GSCs of adult mouse testis, and its’ targeted disruption leads to the complete loss of germ cells in both sexes (Tsuda et al., 2003). Human NANOS3 mutation results in premature ovarian insufficiency (Wu et al., 2013). However, zebrafish homologous gene *nanos3* is expressed in oocytes, but *nanos3* mutation leads to loss of ovarian GSCs and sex-reversal, suggesting that *nanos3* is essential for the maintenance of ovarian GSCs in zebrafish (Draper et al., 2007; Beer and Draper, 2013). *nanos2* is expressed in the spermatogonia at the earliest stage of the mouse testis and is required for maintaining SSCs. *nanos2* maintains its long-term stem cell state by inhibiting the specialization of GSC (Sada et al., 2009). However, *nanos2* is expressed in early GSCs of zebrafish ovary and testis (Beer and Draper, 2013; Cao et al., 2019). Like *nanos3*, *nanos2* mutations lead to loss of GSCs in zebrafish (Figure 1G), suggesting partially redundant roles of *nanos2* and *nanos3* in the maintenance of female GSCs (Cao et al., 2019). Furthermore, *nanos2* mutation led to the defect of ovarian regeneration (Figure 1H) and continuous overexpression of *nanos2* rescued defective ovary regeneration (Cao et al., 2019). Thus, we speculate that ovarian regeneration is also defective in *nanos3* mutants, but it needs further verification.

#### Wnt Signaling

Many reports show that Wnt signaling participates in mammalian gonadal development. Wnt4 homozygous mutant XX mouses have male characteristics, and the loss of oocyte starts to occur, indicating that Wnt4 is necessary for ovary development (Vainio et al., 1999). Moreover, Wnt4 can upregulate the gene nuclear receptor subfamily 0 group B member 1 (NR0B1) and restrict expression of the SRY mammalian male-determining gene (Jordan et al., 2001). Wnt/β-catenin is capable of restricting expression of NR5a1 and Sox9 and the canonical Wnt signaling pathway ligand, Rspo1, is a determinant of mammalian ovary development (Lau and Li, 2009). β-catenin can also regulate expression of gonadal aromatase Cyp19a1a through its interaction with NR5a1 in rat granulosa cells (Parakh et al., 2006). Recent evidences shows that Wnt signaling plays an important role in teleost reproductive development. The specification of zebrafish gonad is regulated by Wnt/β-catenin pathway. Wnt signaling inhibition causes the increase in the proportion of the male and *cyp19a1a* expressed in granulosa cells may be the target gene of Wnt signaling. These results show that Wnt signaling is very important for ovarian development in zebrafish (Sreenivasan et al., 2014). Additionally, Wnt signaling is also reported to be involved in germ cell regeneration. Our study shows that Wnt signaling is activated after ovarian amputation and inhibition of Wnt signaling impairs ovarian regeneration by reducing proliferation of GSCs (Figure 1E) (Cao et al., 2019).

#### Germ Cell-Specific Factors

Many factors were identified to be germ cell-specific genes and involved in germ cell specification and maintainace in zebrafish. These molecular factors are also primarily conserved and may contribute to germ cell regeneration. *vasa* and *dnd* are commonly used as germ cell specific markers. *vasa* is required for germ cell differentiation and GSC maintenance (Hartung et al., 2014). Loss of *dnd* lead to failure of PGC migration and survival (Slanchev et al., 2005). *piwil1* and *piwil2*, the Piwi homologs in zebrafish, are expressed in germ cells. *piwil1*, namely *ziwi*, is essential for germ cell maintenance while *piwil2*, namely *zili*, is required for germ cell differentiation and meiosis (Houwing et al., 2007, 2008). azoospermia-like (*dazl*) is expressed in germ cells and its’ mutation leads to failure of GSC establishment and fertility (Bertho et al., 2021). Based on the fuctions of these molecular factors, we speculate that they may contribute to germ cell regeneration, but it needs further verification.

## Conclusion and Perspectives

In conclusion, progress have been achieved in germ cell regeneration by using zebrafish as an animal model and contributed to the development of this field. However, there are still many unanswered questions in the cellular and molecular mechanisms underlying germ cell regeneration. For example, there is little information on what other cells are involved in the regulation of germ cell regeneration except for GSCs. Furthermore, the interaction between somatic cells and germ cell regeneration, as well as the molecular mechanisms regulating self-renewal and differentiation of GSCs and reproductive regeneration, are still poorly understood. In addition, what factors of injury environment stimulate GSCs behaviors is worth to be explored during regeneration. To answer these questions, some of sequencing technologies such as single cell sequencing and epigenetic testing, which include DNA methylation, histone modification and micro-RNA, will need to be utilized for regeneration studies. Moreover, advances in live imaging technology for adult zebrafish will also help expand our toolbox for germ cell regeneration. With the development of these new technologies, important discoveries in the mechanisms of germ cell and reproductive regeneration using the zebrafish model will be realized. An in-depth understanding of the mechanisms underlying germ cell regeneration in species with highly regenerative abilities such as zebrafish may provide inspiration for therapeutic strategies in mammalians reproductive disease.

## Author Contributions

ZC conceived and carried out the literature review research, designed the figures and diagrams, and wrote the manuscript. QY conceived and carried out the literature review research. LL conceived and carried out the literature review research and wrote the manuscript. All authors contributed to the article and approved the submitted version.

## Conflict of Interest

The authors declare that the research was conducted in the absence of any commercial or financial relationships that could be construed as a potential conflict of interest.
